# Entitlement and me: problems in Canadian medical education

**Published:** 2016-10-18

**Authors:** Lester Liao

**Affiliations:** Department of Pediatrics, University of Alberta

When I was admitted to medical school in Canada, I was commended by my new professors as one of the country’s brightest students. The first months inculcated and cultivated the idea that I was exceptional and set apart from other students who did not make it to medical school. Sometimes the praise was explicit, sometimes not; few probably noticed. Yet there was a subtle message: I was special and deserved all my success because of it.

I have to admit that initially such praise was quite pleasant. Who does not love to be praised? Yet as I contemplated my “greatness” I readily recalled peers who never entered medical school, but were surely just as, if not more, capable than I. We all can remember these students; the approbation helps me forget them.

In his book *Excellent Sheep*, William Deresiewicz shares a colleague’s reflection on the “old days” at Yale.

In… September 1957… I remember the Dean of Yale College telling us that the pool of applicants from which we had been drawn was so large and so good that Yale could have recruited a class every bit as qualified as ours… He went on to say that it was the duty of each of us over the next four years to prove that Yale had made the right choice by picking us ….^1^

He continues to highlight how that mindset changed just over a decade later and students were told they were “the most wonderful set of human beings who had ever entered Yale, and how wonderful it was for Yale that they had decided to attend.”^1^

In Canada today, we fit the latter cohort. Our egos are stroked and we lap it up. This exacerbates the pervasive culture of entitlement. Compared to previous generations, we think far more highly of ourselves.^2^ We believe that we should get what we want and expect to be protected.^3^ This is bolstered by assurances that failing is unlikely (because the school will do everything to prevent you from dropping out) and that second chances are seemingly inexhaustible. Even the white coat ceremony can cultivate our entitlement.^4^ Yet we are so very blind to this. I recall a residency interview when candidates were asked about how the entitlement culture affects medical learners today; nobody had any idea what to say. As a result, I picked up a book on the generation that lived through the Great Depression and World War II. I was chastened. Despite their tremendous sacrifices and accomplishments, they “made no demands of homage” and expected little to be done for them.^5^ Not my generation.

My concerns are not solely polemical or nostalgic. There are tangible, real, and serious negative, if unintended, consequences of these well-meaning accolades. Carol Dweck’s research on fixed and growth mindsets provides insight. The fixed mindset believes, “that your qualities are carved in stone… [it] creates an urgency to prove yourself.”^6^ The growth mindset believes, “that your basic qualities are things you can cultivate through your efforts.”^6^ Dweck’s research suggests that the praise of our intelligence promotes the fixed mindset so we will strive to appear brilliant and avoid situations that might challenge our bloated self-concepts (i.e. our colleagues who cannot publicly accept being wrong about a medical question). This mindset feeds our narcissism, which is detrimental to teamwork and inflates our sense of competence.^2^ In contrast, a growth mindset founded on hard work suggests that many people have the potential for medical school, which lowers our hallowed status.

The problem extends beyond appearing smart. We expect to control “when, where, how, and how fast [we] learn.”^7^ When we encounter a nasty preceptor we decry poor learning conditions and fail to consider that we will inevitably meet and need to learn to work with similar characters. We need resilience for our own health, and this can be encouraged through developing perseverance and responsibility.^8^,^9^ This current paradigm also does not help us consider others’ perspectives.^10^ Our altruistic impulse diminishes when we are over-indulged.

Entitlement is apparent in daily banter regarding personal gain. Lifestyle factors dominate conversations. Of course, we are still taught how to appear “professional,” but this amounts to superficial performance, a mere façade of altruistic character. Furthermore, we believe we deserve prestige and high pay, and hence, performance for these ends dominates our pursuits over the poorly remunerated altruism. Entitlement is changing the profession, and the educational system is fostering it.

I must highlight that these are general observations, and many educators and learners do not fit this broad picture. Mentors have actually developed my awareness of this problem. In addition, the aims of the whole system are surely well-intentioned. I am in favour of encouragement and nurturing; they just need the proper context, imbued with personal responsibility and gratitude. We are currently misguided.

So what now? The overall situation is resistant to change. The education system is driven by learners, and we love to be applauded. The solution begins through both formal and informal means. Formally, we must challenge learners to recognize their own sociocultural influences through studies in the humanities. We should review the ideologies of the medical profession and demands of the physician in every era. Brief seminars on prevalent philosophies and contemporary sociological trends can draw attention to our generational biases. Research that examines negative modern trends, such as Jean Twenge’s work on narcissism,^2^ can build on the studies that simply increase awareness and help us see that change is needed. These will humble us and mitigate what C.S. Lewis called “chronological snobbery,” defined as “the uncritical acceptance of the intellectual climate common to our own age and the assumption that whatever has gone out of date is on that account discredited.”^11^ These initiatives will confront the current underlying entitlement mindset and take us beyond pleasing our teachers with simple external conformity to professional standards.

Informally, learners should be mentored by elder physicians who are able to identify our blind spots and provide experiential wisdom. Entitlement may be nurtured in daily interactions with preceptors, so educators must examine their own attitudes towards medicine and adopt the mindset that learners need to be refined and encouraged, not praised and coddled. Hard work, adaptability to tough situations (as opposed to conforming situations to ourselves), commitment, and a sense of gratitude must be commended.

We must change for our sakes and for those of our patients.
